# Genome-Wide Association Mapping Unravels the Genetic Control of Seed Vigor under Low-Temperature Conditions in Rapeseed (*Brassica napus* L.)

**DOI:** 10.3390/plants10030426

**Published:** 2021-02-24

**Authors:** Tao Luo, Yuting Zhang, Chunni Zhang, Matthew N. Nelson, Jinzhan Yuan, Liang Guo, Zhenghua Xu

**Affiliations:** 1 MOA Key Laboratory of Crop Ecophysiology and Farming System in the Middle Reaches of the Yangtze River/College of Plant Science and Technology, Huazhong Agricultural University, Wuhan 430070, China; luotao28@webmail.hzau.edu.cn (T.L.); zhangchunni@webmail.hzau.edu.cn (C.Z.); yuanjinzhan@mail.hzau.edu.cn (J.Y.); 2 National Key Laboratory of Crop Genetic Improvement, Huazhong Agricultural University, Wuhan 430070, China; ytzhang@webmail.hzau.edu.cn (Y.Z.); guoliang@mail.hzau.edu.cn (L.G.); 3 CSIRO Agriculture and Food, Floreat, WA 6014, Australia; matthew.nelson@csiro.au; 4 The UWA Institute of Agriculture, The University of Western Australia, Perth, WA 6009, Australia

**Keywords:** rapeseed, seed vigor, low-temperature stress, GWAS

## Abstract

Low temperature inhibits rapid germination and successful seedling establishment of rapeseed (*Brassica napus* L.), leading to significant productivity losses. Little is known about the genetic diversity for seed vigor under low-temperature conditions in rapeseed, which motivated our investigation of 13 seed germination- and emergence-related traits under normal and low-temperature conditions for 442 diverse rapeseed accessions. The stress tolerance index was calculated for each trait based on performance under non-stress and low-temperature stress conditions. Principal component analysis of the low-temperature stress tolerance indices identified five principal components that captured 100% of the seedling response to low temperature. A genome-wide association study using ~8 million SNP (single-nucleotide polymorphism) markers identified from genome resequencing was undertaken to uncover the genetic basis of seed vigor related traits in rapeseed. We detected 22 quantitative trait loci (QTLs) significantly associated with stress tolerance indices regarding seed vigor under low-temperature stress. Scrutiny of the genes in these QTL regions identified 62 candidate genes related to specific stress tolerance indices of seed vigor, and the majority were involved in DNA repair, RNA translation, mitochondrial activation and energy generation, ubiquitination and degradation of protein reserve, antioxidant system, and plant hormone and signal transduction. The high effect variation and haplotype-based effect of these candidate genes were evaluated, and high priority could be given to the candidate genes *BnaA03g40290D*, *BnaA06g07530D*, *BnaA09g06240D*, *BnaA09g06250D*, and *BnaC02g10720D* in further study. These findings should be useful for marker-assisted breeding and genomic selection of rapeseed to increase seed vigor under low-temperature stress.

## 1. Introduction

Due to substantial progress in breeding and cultivation practices, rapeseed has become the second most produced oilseed behind soybeans [1,2]. Rapeseed is a major over-wintering crop in the Yangtze River Basin, accounting for more than 80% of national total production in China [3]. A period of exposure to low temperature in the vegetative stage is necessary for rapeseed to achieve cold acclimation and fulfill the vernalization requirement [4,5]. However, the germination and seedling emergence stages of rapeseed are sensitive to temperatures below approximately 10 °C [6,7]. Low temperature is the major environmental stress that narrows the window of success for direct-seeding and limits the geographic distribution of rapeseed in this region. Canada also faces suboptimal temperature conditions when sowing rapeseed, and possibly also in Northern Europe where spring varieties are grown. Seed vigor has been described as the potential for rapid germination and high seedlings developmental rate under a wide range of environmental conditions [8]. Rapid germination and uniform seedling emergence increase the likelihood of stable yield production of rapeseed in highly unpredictable environments and can be achieved through genetic improvement of seed vigor under low-temperature conditions [9].

Germination and seedling emergence are complex multi-step processes involving a series of coordinated physiological and biochemical initiations. The rate of water uptake slows down with decreasing temperature in the initial phase of seed imbibition [10]. Successful seed germination are closely associated with the balance between internal reactive oxygen species (ROS) contents and the activities of ROS-scavenging systems [11,12]. The additional ROS induced by low temperature can disrupt cell homeostasis, thereby hindering the germination process and subsequent seedling establishment. There is wide genotypic variation of low-temperature tolerance for rapeseed genotypes in seed germination and seedling emergence stages [13,14]. The high-vigor seeds showed high levels of late embryogenesis abundant (LEA) protein and aquaporin under low-temperature stress, contributing to the hydraulic activity of cells and re-establishment of metabolisms [15,16]. Seed vigor can also be enhanced by improving the activities of enzymatic (superoxide peroxidase, catalase, and dismutase) and non-enzymatic antioxidants (ascorbic acid, glutathione) [17,18].

Genetic resources are potentially useful for breeding varieties with improved low-temperature tolerance during germination and seedling emergence stage. The genome-wide association study (GWAS) approach is a powerful tool to correlate target traits and genetic markers within a population arising from linkage disequilibrium [19]. With the advent of cost-effective genotyping technologies, GWAS has been widely used in rapeseed, mainly focusing on flowering time [20,21], yield components [22,23,24], and abiotic stress [25,26]. Several promising positional and functional candidate genes have been associated with seed germination speed and vigor under optimal conditions for rapeseed [27]. However, there is still a lack of knowledge regarding the genetic control of rapeseed seed germination and seedling-emergence-related traits under suboptimal conditions including low temperature stress.

The present study used a panel of 442 inbred lines of rapeseed (*B. napus*) collected from different geographic locations. The objectives of this study are (1) to evaluate the low-temperature stress tolerance indices of rapeseed genotype population at the germination and seedling emergence stages; (2) to detect the genetic basis of low-temperature tolerance related marker-trait associations by GWAS; (3) to identify candidate genes potentially involved in the genetic regulation of low-temperature tolerance in rapeseed. The results of this study could benefit the target of breeding rapeseed variety with fast germination and uniform seedling establishment under low-temperature stress.

## 2. Results

### 2.1. The Phenotypic Performance of Rapeseed Accessions to Low-Temperature Stress during Germination and Seedling Emergence Stages

**Figure 1:**
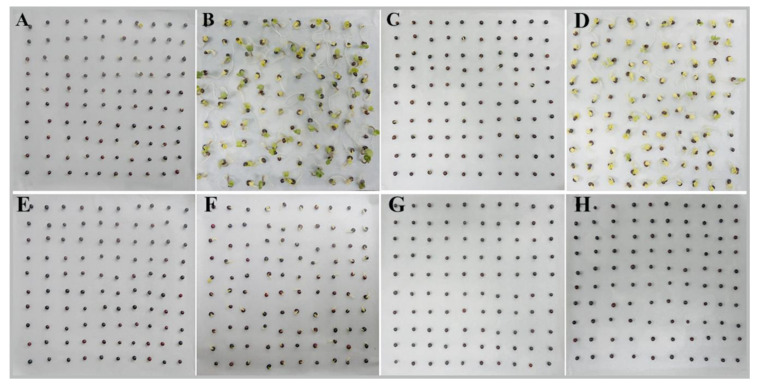
The germination progresses of a strong low-temeprature tolerance genotype ((**A**,**B**), normal temperature for 1 day after imbibition (DAI) and 3 DAI; (**E**,**F**), low temperature for 1 DAI and 3 DAI) and a weak low temperature tolerance genotype ((**C**,**D**), normal temperature for 1 DAI and 3 DAI; (**G**,**H**), low temperature for 1 DAI and 3 DAI).

**Figure 2:**
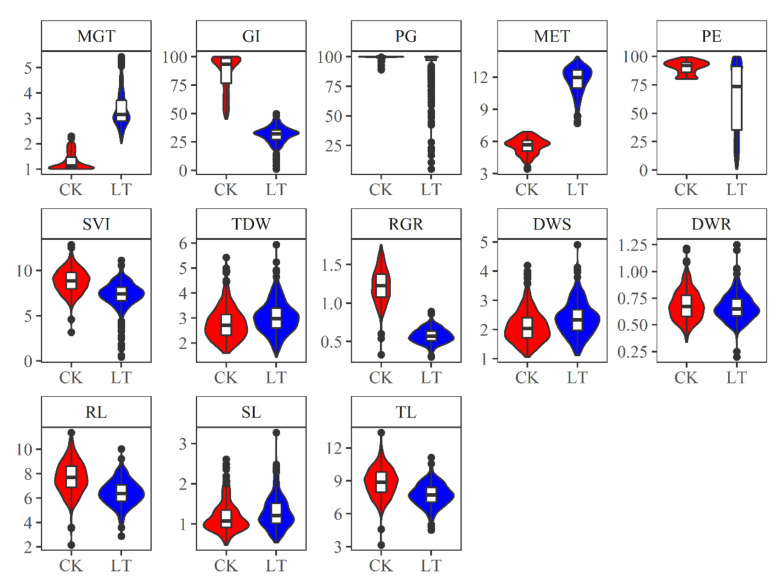
The distribution of germination and seedling emergence indices of 442 rapeseed accessions under normal (CK) and low-temperature stress (LT) conditions. The minimum, the maximum, the sample median, and the first and third quartiles of each index are shown by the boxplot. The probability density of each index is shown by the violin plot. MGT, mean germination time (d); GI, germination index; PG, percentage of germination (%); MET, mean emergence time (d); PE, percentage of emergence (%); DWS, dry weight of shoot (mg plant^−1^); DWR, dry weight of root (mg plant^−1^); TDW, total dry weight (mg plant^−1^); RL, root length (cm); SL, shoot length (cm); TL, total length (cm); SVI, seedling vigor index; RGR, root growth rate (cm day^−1^).

A panel of 442 rapeseed accessions were evaluated to assess the phenotypic traits related to germination and seedling emergence under normal and low-temperature conditions. There is an obvious phenotypic variation among genotypes in responding to normal and low-temperature conditions, an example of which is shown in Figure 1. The distribution of germination and seedling emergence indices of these accessions is presented in Figure 2. Under normal temperature conditions (25/20 °C, etc.), there was little variation of PG and PE among these accessions, ranging from 89% to 100% and from 80% to 99%, respectively. MGT and MET traits ranged from 1.00 to 2.33 d with a mean value of 1.28 d, and from 3.43 to 6.93 d with a mean value of 5.59 d, respectively. The high germination percentage under normal conditions indicated that the seeds have high germination potential without dormancy. Low-temperature stress extended the time to complete germination and seedling emergence processes and reduced the germinant and emerged seed number. Under the low-temperature condition, the PG and PE traits varied from 5% to 100% and from 1% to 100%, respectively. MGT and MET traits ranged from 2.01 to 5.43 d with a mean value of 3.33 d, and from 7.69 to 13.90 d with a mean value of 11.73 d, respectively. Seedling emergence is a heterotrophic growth basing on the seed’s stored energy reserves. The variation of DWS, DWR, and TDW are mainly derived from seed reserves and showed no significant difference between normal and low-temperature conditions. The distribution of RL, SL, and TL among the accessions was similar under normal and low-temperature conditions. Low-temperature stress slowed down root growth rate to an average value of 0.57 cm day^−1^ compared with that under normal condition of 1.23 cm day^−1^. The GI and SVI markedly decreased under low-temperature conditions.

### 2.2. Low-Temperature Stress Tolerance Indices during Germination and Seedling Emergence Stages

**Figure 3:**
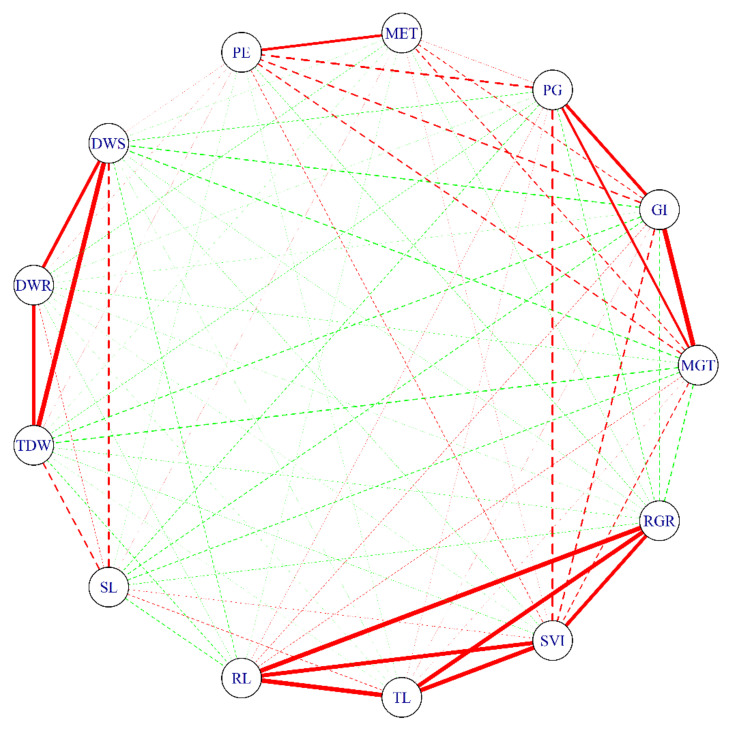
The correlation network for the low temperature stress tolerance indices during the germination and seedling emergence stages based on 442 rapeseed accessions. The two variables connected by the red line are positively correlated (0 < r ≤ 1), and green lines indicate negative correlation (−1 ≤ r ≤ 0) between the two variables. The correlation value was represented by the width of the line. Solid lines indicate a large correlation value (r > 0.5). MGT, mean germination time; GI, germination index; PG, percentage of germination; MET, mean emergence time; PE, percentage of emergence; DWS, dry weight of shoot; DWR, dry weight of root; TDW, total dry weight; RL, root length; SL, shoot length; TL, total length; SVI, seedling vigor index; RGR, root growth rate.

Low-temperature stress tolerance was estimated by the stress tolerance indices (STIs) for different traits regarding seed germination and seedling emergence under normal and low-temperature conditions. The traits related to seed germination and seedling emergence showed different variations for low-temperature tolerance. The STI of SL has the highest genotypic coefficient of variation of 0.58, and the STI of RGR had the lowest genotypic coefficient of variation of 0.10 (Table 1). The estimated broad-sense heritability of germination- and seedling-emergence-related indices ranged from 0.88 to 0.99 among the traits of interest (Table 1). Correlation network analysis was performed to further investigate the relationship among the STIs of different traits (Figure 3). A strong correlation structure existed among RL, TL, SVI, and RGR; among GI, PG, and MGT; between PE and MET; and among DWS, DWR, and TDW traits. Principal component analysis (PCA) was conducted to diminish the redundancy of correlated multivariate traits and integrated a few key indicators to reflect low-temperature tolerance. As presented in Table 2, the STIs of RL, TL, SVI, and RGR had a high loading to PC1, which reflected the low-temperature tolerance on traits of seedling morphology. The STIs of GI, PG, and MGT had a high loading to PC2, which reflected the low-temperature tolerance on traits of fast germination speed and high germination rate. The STIs of DWS, DWR, and TDW had a high loading to PC3, which reflected the low-temperature tolerance on traits of plant biomass. The STIs of PE and MET had a high loading to PC4, which reflected the low-temperature tolerance on traits of fast emergence speed and high seedling emergence rate. The STI of SL individually had a high loading to PC5.

### 2.3. Association of SNP Markers and Low-Temperature Tolerance Indices by GWAS

**Figure 4:**
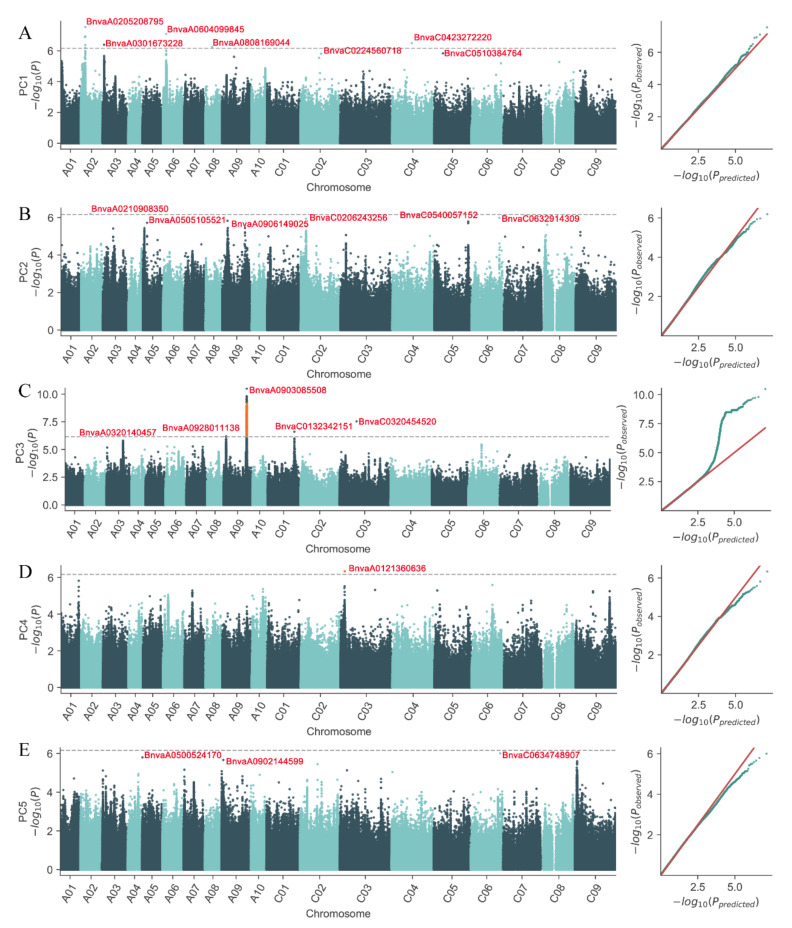
The Manhattan plot of individual marker analysis *p*-value and quantile-quantile (QQ) plot of quantile distribution for 442 rapeseed accessions. The y-intercept of horizontal dash lines indicate the −log_10_ (p) significance threshold. The lead SNPs are marked in red and named according to the chromosome and the position of chromosome. For example, BnvaC0224560718 indicates the SNP is anchored on the physical position of 24560718 bp in chromosome C02. PC1 is the comprehensive STI index of RL, TL, SVI, and RGR (**A**). PC2 is the comprehensive STI index of GI, PG, and MGT (**B**). PC3 is the comprehensive STI index of DWS, DWR, and TDW (**C**). PC4 is the comprehensive STI index of GI, PG, and MGT (**D**). PC5 is the STI index of SL (**E**). MGT, mean germination time; GI, germination index; PG, percentage of germination; MET, mean emergence time; PE, percentage of emergence; DWS, dry weight of shoot; DWR, dry weight of root; TDW, total dry weight; RL, root length; SL, shoot length; TL, total length; SVI, seedling vigor index; RGR, root growth rate.

More than 8 million SNPs (single-nucleotide polymorphisms) were identified across the accessions to provide the genotype dataset for the GWAS analysis. The five integrated traits (principal components) related to seed germination and seedling emergence were used as phenotypic data to detect significant low-temperature tolerance QTLs. Normal or nearly normal distributions were observed for low-temperature stress indices of principal components in the mapping population (Figure S1). The QQ plots in Figure 4 show that the observed *p*-value matched the uniform distribution initially, and eventually diverged from the expected *p*-value, indicating a deviation caused by selection pressure. Manhattan plots illustrate the distribution of marker-trait associations across the genome and highlight some regions that are significantly associated with tolerance to low temperature (Figure 4). The threshold level was determined at a significant *p*-value of 6.91 × 10^−7^ for principal components. In total, 22 QTLs were significantly associated with the integrated traits related to low-temperature stress tolerance indices (Table 3), in which 7 QTLs were associated with PC1 traits, followed by 6 QTLs to PC2, 5 QTLs to PC3, 1 QTL to PC4, and 3 QTLs to PC5. There were 13 QTLs localized in chromosomes A01, A02, A03, A05, A06, A08, and A09 and 9 QTLs localized in chromosomes C01, C02, C03, C04, C05m and C06 (Figure 4).

### 2.4. Candidate Gene Prediction

The candidate genes were sought in the 150 kb flanking regions of each significantly associated SNP locus. According to the high association of SNPs and gene function, 62 candidate genes related to seed vigor are identified (Table 3). The majority of predicted candidate genes could be divided into DNA repair and RNA translation, mitochondrial activation and energy generation, ubiquitination and degradation of reserve, antioxidant system, and plant hormone and signal transduction.

Hydraulic proteins and chaperone such as LEA, aquaporin and heat shock protein were detected at PC1 and PC2 trait-associated loci, potentially contributing to the structural stability and functional integrity of proteins under low water potential conditions, especially at the initial imbibition stage. Germination and seedling morphogenesis are driven by heterotrophic growth based on the seed’s stored reserves. Five pentatricopeptide repeat-containing proteins, one mitochondrial import inner membrane translocase subunit, and one mitochondrial substrate carrier family protein located in mitochondrion homologues were detected as potential genes participating in the mitochondrial biogenesis and activation. Three genes, *BnaC04g22140D*, *BnaA03g03560D*, and *BnaA02g18190D* related to oxidative phosphorylation to synthesize ATP were associated with PC1 and PC2 traits, resulting in fast germination speed and high seedling vigor. A pyruvate kinase family protein gene (*BnaC03g33590D*) involved in glycolysis/gluconeogenesis pathway and a key gene *BnaA03g40170D* in pentose phosphate pathway were significantly associated with PC3 traits. Three auxin biosynthesis and responsive related genes indole-3-pyruvate monooxygenase, auxin-like 1 protein and SAUR-like auxin-responsive protein homologues appeared to contribute to PC5 traits. The gibberellin-regulated protein gene (*BnaC05g42910D*), ethylene-responsive transcription factors (*BnaA02g10340D*, *BnaA03g40380D* and *BnaA09g39350D*), and dehydration-responsive protein homologues genes (*BnaA09g04520D* and *BnaA01g31290D*) also appeared to play important roles at different stages during seed germination and seedling emergence.

**Figure 5:**
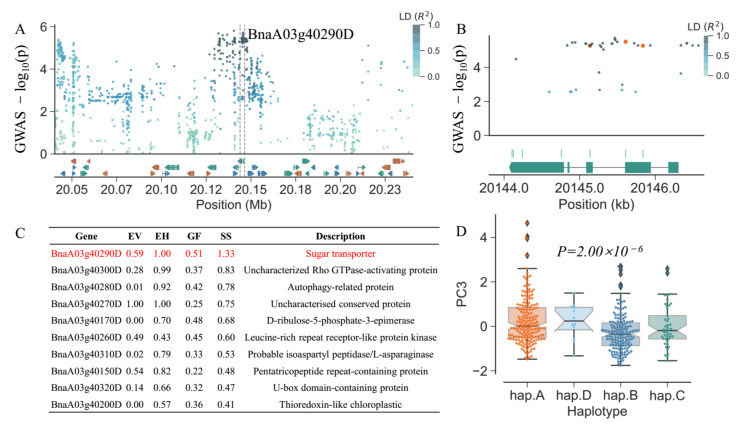
Prioritization of candidate gene for SNP BnvaA0320140457 based on comprehensive score of GWAS *p*-value, large effect variation and haplotype variation. (**A**) Manhattan plot of the SNPs in the 150 kbp regions flanking the candidate gene *BnaA03g40290D*, and the window of the dash lines represent the SNPs located in the region of the specific gene. (**B**) GWAS results at the *BnaA03g40290D* gene itself with high effect SNPs are indicated by gold circles. (**C**) Ranking the top ten genes according to the summary score and the top-ranked gene is the most likely casual gene. EV, the effect of variations; EH, effect of haplotypes; GF, the score of probability for gene function; SS, summary score of GF, EV, and EH. (**D**) Box plots displaying the impacts of haplotypes variations derived from the specific gene on the phenotype.

We applied a systematic candidate gene scoring function to evaluate the internal variation, known function, and haplotype effects of these candidate genes. The casual genes in the top ranking (summary score > 1) were *BnaA03g40290D* (Figure 5), *BnaA06g07530D* (Figure S2), *BnaA09g06240D* (Figure S3), *BnaA09g06250D* (Figure S4), and *BnaC02g10720D* (Figure S5). *BnaA06g07530D* encodes a RING/U-box superfamily protein involved in protein ubiquitination. *BnaC02g10720D* and *BnaA09g06250D* encode a peroxidase superfamily protein responding to oxidative stress. *BnaA03g40290D* and *BnaA09g06250D* encode sugar transport proteins located in mitochondrion to participant in carbohydrate transmembrane transporter activity. As shown in Figure 5B, we found seven missense variations in *BnaA03g40290D*. Meanwhile, according to a set of neighboring SNPs in the gene region and upstream 2 kb region, *BnaA03g40290D* was grouped into four haplotypes with a significant difference in phenotypic means (*p* = 2.00 × 10^−6^; Figure 5D). Based on these results, we believe that these candidate genes should be further investigated for their potential role in seed vigor in rapeseed.

## 3. Discussion

Poor or non-uniform seedling establishment of rapeseed cultivars due to low-temperature stress is one of the major challenges of rapeseed production in the Yangtze River Basin, especially under late direct-seeding conditions. The present study surveyed the traits regarding the germination and seedling emergence process in a large panel of rapeseed accessions, which provides valuable germplasm resources information for biodiversity. In our study, the low-temperature regime extended the MET and MGT more than twofold compared with the optimal temperature. Fast-germinating seed may increase the probability of successful emergence and competitive success in crop establishment [28]. Fast-germinating genotypes are accompanied by a higher rate of germination; this link was also applied to the performance of seedling emergence speed and final emergence percentage. When a seed germinates, the radicle is the first organ to come out and to elongate to form the primary root. A well-developed root system is crucial for absorbing water and mineral salts from the soil [29]. Low temperature in our study retarded the average root growth rate to half of that under normal conditions. The root growth rate showed a high degree of variability among the genotypes under normal temperature conditions, while the percentage of germination and seedling emergence exhibited a high degree of variability under low-temperature conditions. This genotypic variability could benefit the selection and improvement of low-temperature tolerance of rapeseed to cope with unpredictable cold weather, especially under late direct-seeding condition in Yangtze River Basin. This principle could also be applied to other rapeseed growing regions that experience cold conditions during sowing such as early sown rapeseed in spring in cold regions of Canada and northern Europe, or late sown rapeseed in autumn in Australia.

It is a breeding target to select varieties that perform well under both optimal and stressed conditions so that they can adapt well to a changing climate [30]. Therefore, the stress tolerance index (STI) was used in this study to quantify performance in both optimal and low-temperature stress conditions. Many functional traits have been defined to capture the fitness of the species to the environment during the germination and seedling emergence process. Combining several correlated traits can give a better prediction of subsequent field performance than any single trait score [31]. The STIs of thirteen traits were evaluated in the germination and seedling emergence process and showed high broad-sense heritability. As seed lots were tested for germination under well-controlled laboratory conditions, the observed differences in STI performance could be largely attributed to genotypic variation. Principal component analysis clustered these STIs into five groups corresponding to specific functions. PC2 describes well the germination ability, while the other principal components (PCs) can describe the performance at the seedling emergence stage. Seed vigor, constituted as the level of activity and performance of the seed lot during germination and seedling emergence, is an important trait to cope with low-temperature stress at the initiation of plant lifecycle [32]. Selection of seed lots on the basis of germination characteristics alone is not necessary to determine successf in seedling establishment [33]. No significant correlations of PC2 with other PCs agreed with the findings that time to radicle protrusion and seedling growth rate contributed independently to seed vigor performance [34].

Seed germination and subsequent seedling emergence are a series of progressive physiological and biochemical processes influenced by both genetic and environmental factors. Different genes have been assumed to play critical roles in the processes of rehydration, anaerobic/aerobic respiration, and stored reserves mobilization during seed germination and seedling emergence [16,35]. With the development of high-throughput SNP genotyping technology, GWAS have been conducted to dissect the SNPs associated with rapid germination and high seedling vigor for rapeseed under normal, salt, and drought stress conditions [27,36]. To estimate the contribution of genotypic variations to the low-temperature stress tolerance indices under seed germination and seedling emergence stage, GWAS was performed in the present study to identify genomic intervals and candidate genes for the five PC traits. Using principal component scores as dependent variables is considered an efficient strategy to perform GWAS as it could decrease the likelihood of a type I error rate, transform the skewed original variables into approximately normal distribution, and detect genomic regions that could be overlooked by using individual traits [37]. These five PCs could act well as comprehensive indicators for seed vigor under low-temperature stress. In total, 22 QTLs are associated with low-temperature tolerance during seed germination and seedling emergence stages, among which which 7 QTLs are associated with PC1 traits, followed by 7 QTLs with PC2, 5 QTLs with PC3, 1 QTL with PC4, and 3 QTLs to PC5. While some of the QTLs detected in PC2, PC4, and PC5 did not exceed the significance threshold, they were close to the threshold and stood out compared with the surrounding SNP markers. To date, the QTL analysis for seed vigor of rapeseed under low-temperature stress is still rare; thus, it is difficult to directly compare with previous reported QTLs. An LD interval harboring QTL for fast germination and germination rate under normal condition in chromosome C6 was mapped by Hatzig et al. (2015) in the vicinity (~78 kb) of SNP BnvaC0632914309 associated with PC2 traits in our study. Wan et al. [38] identified an SNP associated with germination rate under salt stress, which was located within ~18kb distance of SNP BnvaA0505105521 associated with PC2 traits in our study. Taken together, these studies provide independent support for the significant role of these genomic regions in seed germination traits under different conditions.

Functional genes around the QTL could provide insights into linking the morphological occurrence processes of seed germination and seedling emergence with molecular mechanism regulated by gene expression and its interaction with the external environmental factors. Sixty candidate genes related to seed germination and seedling emergence were detected within 150 kb upstream and downstream of different significant markers, mainly involving in the DNA repair and RNA translation, mitochondrial activation and energy generation, ubiquitination and degradation of protein reserve, antioxidant system, and plant hormone and signal transduction. For germination to occur rapidly, quiescent seeds need to quickly activate the enzymes and functional proteins required to resume metabolism and to initiate cellular events that lead to radicle emergence. Late embryogenesis abundant (LEA) proteins and heat shock proteins (HSPs) are intensively synthesized as a part of the embryogenesis program and exerted protective molecules in dehydration process during seed maturity [39]. Proteomic analysis revealed that differential accumulations of LEA proteins and HSPs in the seed maturation phase were speculated to cause the discrimination in seed vigor and longevity [40,41]. Homologues of the *A. thaliana* HSP genes *AT5G12020* and *AT5G10680* were associated with PC1 traits, and homologues of LEA proteins genes (*AT5G53820* and *AT5G53730*) were associated with PC2 traits (Table 3). These hydraulic proteins and chaperones may help improve structural stability and maintain the function of proteins under low-temperature conditions, especially at the initial imbibition stage in rapeseed.

The regulation of stored mRNA associated with protein synthesis is considered to be an essential determinant of seed vigor when switching from desiccation to imbibition [42,43]. The majority of these residual mRNAs encoded by seed maturation genes are gradually degraded following imbibition and replaced by de novo synthesized transcripts. During early seed imbibition, the primary step was to repair the DNA damage accumulated in the embryo of seeds [44], and the DNA mismatch repair protein gene *AT4G17380* associated with PC1 and DNA gyrase subunit gene *AT5G04130* associated with PC2 may be involved in this process. The RNA helicase genes (*AT5G26742* and *AT3G62310*), RNA-binding protein gene *AT1G13190*, and 3′-5′-exoribonuclease genes *AT3G61620* genes enriched in mRNA surveillance pathway mediated the quality control mechanism by detecting and degrading abnormal mRNAs [45,46,47]. The 40S ribosomal protein gene *AT5G58420* and Aminoacyl-tRNA biosynthesis-involved genes (*AT3G61690* and *AT5G26830*) could play an important role in the translational regulation to catalyze protein synthesis during seed germination and seedling transition [48,49]. The abundance in polysomal mRNA isolated from total mRNA revealed a timely regulated and selective recruitment of mRNAs for translation during seed germination in *A. thaliana* [50]. Increasing the ribosomal protein gene expression and ribosomal activity is an early germination-associated event to facilitate the de novo synthesis of proteins [51]. Vigorous seed must rapidly remobilize stored reserves to provide nutrients for the post-germination events before it transits to autotrophic metabolism. Efficient utilization of stored reserves to provide new products for energy demand and morphological construction could increase seedling dry weight accumulation [34]. The endo-beta-mannosidase gene *AT3G10890* associated with PC4 traits is required for the breakdown of galactomannans in seed [52]. It has been reported that endo-beta-mannosidase increased activity during seed imbibition and participated in the mobilization of the mannan-containing cell walls of seed endosperm in tomato [53]. The stored proteins are converted to amino acids predominantly by the ubiquitin-proteasome system [54], and the related E3 ubiquitin-protein ligase gene, ubiquitin carboxyl-terminal hydrolase protein gene, and protease gene were detected in our study. The D-ribulose-5-phosphate-3-epimerase gene *AT5G61410* and 3-phosphoglycerate kinase gene *AT5G61450*, both located on the flanking region of the lead SNP BnvaA0320140457 associated with PC3 traits, are well known to regulate the energy supply involved in pentose phosphate pathway and glycolysis, separately [55,56]. The pentatricopeptide repeat-containing proteins are RNA binding proteins involved in post-transcriptional processes in mitochondria and chloroplasts [57,58]. Five pentatricopeptide repeat-containing protein genes were detected and putatively targeted to the mitochondria, which may play important roles in mitochondrial biogenesis.

Exposure to low temperature is expected to trigger the generation of early signals such as increasing intracellular Ca^2+^ and secondary signaling molecules such as inositol phosphate and reactive oxygen species (ROS) as well as activation of kinase cascades [59]. ROS have a dual role in seed physiology, and excessive ROS accumulation can lead to the oxidative destruction of cells and organelles [60,61]. The activity of the ROS-scavenging system was increased to alleviate ROS toxicity in cells and organelles in a fast-germinating genotype under low-temperature stress [15]. The candidate thioredoxin superfamily protein gene *AT1G21350* and peroxidase gene (*AT5G58390* and *AT5G62810*) take an active part in scavenging ROS and maintaining the intracellular redox status for successful germination and seedling emergence, especially under low-temperature stress [62,63]. It has been well elucidated in several plants that the AP2/ERF family transcript factors DREB (dehydration responsive element binding) and ERF (ethylene-responsive element-binding factor) play vital roles in regulating the diverse stress responses through the modulation of several signaling pathways [64,65]. Two DREB (*AT3G11020* and *AT5G25610*) and three EFR (*AT5G53290*, *AT5G61600*, and *AT3G61630*) transcript factor homologues were predicted to regulate low-temperature tolerance during germination and seedling emergence stages in this study (Table 3). Meanwhile, a 1-aminocyclopropane-1-carboxylate synthase (ACS) gene *AT3G49700* has been detected to mediate the rate-limiting step in ethylene biosynthesis during seed germination [66,67], which is in accordance with the previous report that ethylene production is associated with abiotic stress in plant [68,69]. Substantial genes near QTLs with unknown functions still need further research to determine their contribution to seed vigor.

Overall, the differences in rapeseed genotypes’ response to low-temperature stress have been evaluated during seed germination and seedling emergence stages. The principal component analysis (PCA) on 13 low-temperature STIs revealed that the first five principal components (PCs) provided the most information on seed vigor under low temperature. Subsequently, the GWAS analysis of low-temperature tolerance was conducted using these 5 PCs and revealed 22 marker-traits-associated SNPs. Sixty candidate genes with known function were identified to be involved in regulation of seed vigor. Based on the comprehensive score of the GWAS *p*-value, large effect variation, and haplotype variation, high priority should be given to the candidate genes *BnaA03g40290D*, *BnaA06g07530D*, *BnaA09g06240D*, *BnaA09g06250D*, and *BnaC02g10720D* in further research to reveal the molecular mechanisms underlying seed vigor. This study can contribute to a better understanding of natural variations related to seed vigor under low-temperature stress and provide a useful genetic resource for breeders targeting seed vigor improvement under low temperatures for rapeseed.

## 4. Materials and Methods

### 4.1. Seed Production

A panel of rapeseed accessions comprising 442 inbred lines originating mostly from China with diverse genetic backgrounds (Table S1) were grown and self-pollinated by enclosing the inflorescences in perforated polyethylene bags before the flowers opened during the growing season of 2016–2017 in Wuhan, China. Agronomic management operations including fertilization, irrigation, insect pesticide, and artificial weeding were consistent for all the plots during the growth period. After harvesting and threshing, the fresh seeds of each accession were stored in a seed-storage cabinet with maintaining temperature to 23 °C and relative humidity to 8% for further use.

### 4.2. SNP Markers Identification

Genomic DNA was extracted from the leaves of each accession at the seedling stage using the TIANGEN plant genomic DNA kit (Tiangen, Beijing, China). A DNA library for each accession was constructed using the TruSeq Library Construction Kit (v2), and paired-end reads (2 × 150 bp) were sequenced on an Illumina Hiseq platform at Novogene Bioinformatics Technology Company (Beijing, China). The average whole-genome resequencing depth of these samples was ~8.7. The reads were aligned to the *B. napus* Darmor-bzh v4.1 [70] by BWA software with command ‘mem -M -k 32 -t 4′ [71,72], and the PCR duplicates of sequencing reads were removed with SAMTools [73]. The Genome Analysis Toolkit (GATK v3.6) was used to identify sequence variations among all the accessions with HaplotypeCaller module and command ‘-T HaplotypeCaller -allowPotentiallyMisencodedQuals -emitRefConfidence GVCF’ [74]. ‘GenotypeGVCFs’ command was used to merge the GVCF files. If the mapping quality was less than 20 or sequencing depth was more than 50 across the whole population, the related SNPs and InDels were filtered out.

### 4.3. Germination Trials

It has been widely reported that the optimum temperature range for seed germination of canola is roughly from 20 to 25 °C, and germination at 10–15 °C was found to be the suitable condition for discrimination among rapeseed genotypes [75,76,77]. The germination trials were conducted under two temperature regimes (25/20 °C for normal temperature treatment; 15/10 °C for low-temperature treatment) with 12 h diurnal white-light condition (PAR:150 μmol photons m^−2^ s^−1^) in plant growth chambers with three replications. Diurnally alternating temperatures simulate the natural day/night temperature fluctuations experienced by germinating seeds in the field. Prior to the germination trial, the intact and uniform seeds were selected and surface-sterilized in 0.1% sodium hypochlorite for 15 min. Afterward, the seeds were rinsed cleanly with running water and allowed to air dry at ambient temperature. For each combination of genotypes, temperature treatments and replications, 100 seeds were sown on three layers of filter paper in a germination box. The filter papers in each germination box were saturated with 10 mL distilled water and replenished daily with 1 mL distilled water during the experimental period to provide adequate water for seed germination. The germinant seeds and emergent seedlings in each germination box were counted once daily. A seed was defined as germinated when the radicle protruded through the seed coat by ~1 mm. Seedling emergence was recorded when two cotyledons had completely flattened and the hypocotyl was upright [13]. The duration of germination tests were 7 d and 14 d under normal and low-temperature conditions, respectively. When the germination trial was terminated, the shoot and root lengths of 10 emergent seedlings were measured in each germination box. The roots and shoots of all seedlings were then harvested separately and dried at 80 °C until reaching a constant weight.

### 4.4. Low-Temperature Tolerance Assessment

The phenotypic traits at the seed germination and seedling emergence stages were calculated by our previous description [13], including mean germination time (MGT), germination index (GI), percentage of germination (PG), mean emergence time (MET), percentage of emergence (PE), dry weight of shoot (DWS), dry weight of root (DWR), total dry weight (TDW), root length (RL), shoot length (SL), total length (TL), and seedling vigor index (SVI). The root growth rate (RGR) was estimated by dividing root length by the duration between 50% germination time and termination of the experiment. The low-temperature stress tolerance indices (STIs) of positive traits were calculated according to the formula: STI = (Ys × Yp)/(Yms × Ymp), wherein Yp and Ys are the index values under normal and low temperature stressed conditions, respectively; Ymp and Yms are the average index value among all the genotypes under normal and low temperature stressed conditions, respectively [78,79]. For the negative traits MGT and MET (larger value is linked to poor performance in the germination and seedling emergence stages), the STIs were calculated by reversing the numerator and denominator of the above equation.

### 4.5. Statistical Analysis

The data of STI traits were subjected to ANOVA using R software [80], and the broad-sense heritability (***h*^2^**) of STI traits were calculated to measure the proportion of genetic factor to phenotypic variance by the formula (Equation (1)):

(1) $$h^{2}=\sigma_{g}{}^{2}/\left(\sigma_{g}{}^{2}+\sigma_{e}{}^{2}\right)$$

wherein $\sigma_{g}{}^{2}$ and $\sigma_{g}{}^{2}$ are the genetic variance and the environmental variance, respectively. Genotypic coefficient of variation (GCV) was calculated as follows (Equation (2)):

(2) $$GCV=\sqrt{\sigma_{g}{}^{2}}/\bar{X}$$

wherein $\bar{X\;}$ is the grand mean for each STI index [81]. R package psych 1.5.8 was used to perform principal component analysis (PCA) for the STI indices to reduce the redundancy of correlated, multivariate data without losing important information. The principal components were determined with eigenvalues higher than 1.

### 4.6. Genome-Wide Association Analysis

After excluding SNP markers with a minor allele frequency (MAF) < 0.05, a total of 8,554,109 SNPs were used for GWAS analysis. Genome-wide linkage disequilibrium (LD) decay was estimated using squared allele frequency (r^2^). In this study, the cut-off threshold of r^2^ was 0.2, which represented an average genome-wide physical distance for LD decay of 48.4 kb (Figure S6). The ancestry kinships (K) analysis in the previous study revealed that these accessions could be divided into three subpopulations. The GEMMA python package was used to test the associations between SNPs and phenotype by using the linear mixed model [82,83]. The significance threshold (6.91 × 10^−7^) of associations was calculated by Genetic Type I error calculator (GEC) software to extend the conventional Bonferroni procedure and control the genome-wide type I error rate at 0.05 [84]. The lead SNP marker, defined as the SNP marker with the smallest *p*-value in the genomic region showing an obvious single hump, was also detected as the significant associated SNP markers. Manhattan plots were constructed to display the significant SNP markers, and the effectiveness and appropriateness of the model were assessed using quantile–quantile (Q–Q) plots. Candidate genes were sought in the 150 kb flanking regions of significantly associated SNP loci, and gene annotations in the selected regions were predicted using the Arabidopsis Information Resource [85]. The most promising candidate genes were determined based on (i) their potential attributions to seed vigor in various environment conditions reported in the scientific literature, and (ii) containing highly associated SNP markers within the coding regions. In order to give a priority to the candidate genes, a comprehensive score was evaluated for each candidate gene by considering the GWAS gene internal and promoter region QTLs *p*-value, effect variation, and haplotype variation [86].

## Figures and Tables

**Table 1:** The statistical description of the low-temperature stress tolerance indices (STI) related to germination and seedling emergence. MGT, mean germination time; GI, germination index; PG, percentage of germination; MET, mean emergence time; PE, percentage of emergence; DWS, dry weight of shoot; DWR, dry weight of root; TDW, total dry weight; RL, root length; SL, shoot length; TL, total length; SVI, seedling vigor index; RGR, root growth rate; GCV, genotypic coefficient of variation; h^2^, broad-sense heritability.

STI	Mean	Genotypic Variance	Environmental Variance	GCV	h^2^
MGT	1.11	0.12	0.005	0.32	0.96
GI	1.02	0.12	0.005	0.34	0.96
PG	1.00	0.02	0.002	0.14	0.92
MET	1.04	0.06	0.002	0.23	0.97
PE	1.01	0.25	0.011	0.50	0.96
DWS	1.04	0.24	0.008	0.47	0.97
DWR	1.01	0.11	0.021	0.33	0.84
TDW	1.03	0.18	0.006	0.42	0.97
RL	1.01	0.08	0.010	0.28	0.88
SL	1.06	0.37	0.013	0.58	0.97
TL	1.01	0.06	0.008	0.23	0.88
SVI	1.01	0.08	0.009	0.28	0.90
RGR	1.01	0.01	0.011	0.10	0.89

**Table 2:** The principal component analysis (PCA) for the stress tolerance indices regarding seed germination and seedling emergence. MGT, mean germination time; GI, germination index; PG, percentage of germination; MET, mean emergence time; PE, percentage of emergence; DWS, dry weight of shoot; DWR, dry weight of root; TDW, total dry weight; RL, root length; SL, shoot length; TL, total length; SVI, seedling vigor index; RGR, root growth rate.

Stress Tolerance Index	Standardized Factor Loadings of Principal Components				
	PC1	PC2	PC3	PC4	PC5
MGT	−0.01	0.89	−0.13	0.12	−0.06
GI	0.02	0.95	−0.11	0.13	−0.06
PG	0.07	0.80	0.00	0.11	−0.03
MET	−0.03	0.05	−0.08	0.91	0.03
PE	−0.01	0.31	0.08	0.82	−0.02
DWS	−0.04	−0.16	0.92	0.04	0.21
DWR	−0.02	0.05	0.89	−0.07	−0.09
TDW	−0.04	−0.13	0.96	0.02	0.16
RL	0.98	0.06	−0.07	−0.01	−0.16
SL	−0.01	−0.12	0.19	0.01	0.97
TL	0.98	0.02	−0.01	−0.01	0.17
SVI	0.89	0.38	0	0.03	0.13
RGR	0.94	−0.26	−0.04	−0.05	−0.15
SS loadings	3.59	2.72	2.64	1.56	1.12
Proportion Explained	0.31	0.23	0.23	0.13	0.10

**Table 3:** Candidate genes related to seed germination and seedling emergence under low-temperature stress. PC1 is the integrated STI index of RL, TL, SVI, and RGR. PC2 is the integrated STI index of GI, PG, and MGT. PC3 is the integrated STI index of DWS, DWR, and TDW. PC4 is the integrated STI index of GI, PG, and MGT. PC5 is the STI index of SL. The SNP is named according to the chromosome and the position of the chromosome. For example, BnvaC0224560718 indicates that the SNP is anchored on the physical position of 24560718 bp in chromosome C02. MGT, mean germination time; GI, germination index; PG, percentage of germination; MET, mean emergence time; PE, percentage of emergence; DWS, dry weight of shoot; DWR, dry weight of root; TDW, total dry weight; RL, root length; SL, shoot length; TL, total length; SVI, seedling vigor index; RGR, root growth rate.

Trait	SNP	Distance	Candidate Gene	Arabidopsis	Annotation
PC1	BnvaC0224560718	12.46	*BnaC02g26760D*	AT4G03000	E3 ubiquitin-protein ligase
PC1		16.84	*BnaC02g26770D*	AT4G05230	Ubiquitin-like superfamily protein
PC1	BnvaC0423272220	−91.32	*BnaC04g22040D*	AT3G60690	SAUR-like auxin-responsive protein family
PC1		11.58	*BnaC04g22140D*	AT3G60330	ATPase plasma membrane-type
PC1	BnvaC0510384764	−10.32	*BnaC05g16590D*	AT1G21350	Thioredoxin superfamily protein
PC1		−9.44	*BnaC05g16600D*	AT1G21350	Thioredoxin superfamily protein
PC1	BnvaA0808169044	−25.69	*BnaA08g08250D*	AT4G17380	DNA mismatch repair protein MSH4
PC1		−14.58	*BnaA08g08260D*	AT4G17380	DNA mismatch repair protein MSH4
PC1	BnvaA0205208795	77.15	*BnaA02g10340D*	AT5G53290	Ethylene-responsive transcription factor CRF3
PC1		−128.01	*BnaA02g10070D*	AT5G53820	Late embryogenesis abundant protein
PC1	BnvaA0301673228	−1.43	*BnaA03g03460D*	AT2G37410	Mitochondrial import inner membrane translocase subunit
PC1		15.69	*BnaA03g03500D*	AT1G05890	E3 ubiquitin-protein ligase ARI5
PC1		29.04	*BnaA03g03560D*	AT5G11770	NADH dehydrogenase [ubiquinone] iron-sulfur protein
PC1		90.01	*BnaA03g03740D*	AT5G12020	17.6 kDa class II heat shock protein
PC1	BnvaA0604099845	−45.85	*BnaA06g07530D*	AT1G13195	RING/U-box superfamily protein
PC1		−44.29	*BnaA06g07540D*	AT1G13190	RNA-binding (RRM/RBD/RNP motifs) family protein
PC1		−43.08	*BnaA06g07550D*	AT1G13190	RNA-binding (RRM/RBD/RNP motifs) family protein
PC1		6.61	*BnaA06g07680D*	AT1G13040	Pentatricopeptide repeat-containing protein
PC2	BnvaA0505105521	114.27	*BnaA05g09450D*	AT2G34390	Aquaporin NIP2-1
PC2		119.64	*BnaA05g09470D*	AT2G34390	Aquaporin NIP2-1
PC2	BnvaA0210908350	102.81	*BnaA02g18190D*	ATCG01250	NADH-Ubiquinone/plastoquinone (complex I) protein
PC2	BnvaA0906149025	−35.34	*BnaA09g11790D*	AT1G63970	2-C-methyl-D-erythritol 2,4-cyclodiphosphate synthase
PC2	BnvaC0540057152	−46.13	*BnaC05g42830D*	AT5G04130	DNA gyrase subunit
PC2		−20.54	*BnaC05g42910D*	AT3G10185	Gibberellin-regulated protein
PC2	BnvaC0632914309	−24.57	*BnaC06g32860D*	AT1G71850	Ubiquitin carboxyl-terminal hydrolase family protein
PC2	BnvaC0206243256	−108.19	*BnaC02g10620D*	AT5G58670	Phosphoinositide-specific Phospholipase C
PC2		−34.26	*BnaC02g10680D*	AT5G58420	40S ribosomal protein S4
PC2		−1.97	*BnaC02g10720D*	AT5G58390	Peroxidase
PC2		7.7	*BnaC02g10730D*	AT5G07070	CBL-interacting serine/threonine-protein kinase
PC2		16.96	*BnaC02g10780D*	AT5G58330	Malate dehydrogenese
PC2		28.27	*BnaC02g10830D*	AT5G58290	26S protease regulatory subunit
PC3	BnvaA0320140457	−81.91	*BnaA03g40170D*	AT5G61410	D-ribulose-5-phosphate-3-epimerase
PC3		−42.95	*BnaA03g40210D*	AT5G61450	3-Phosphoglycerate kinase
PC3		4.76	*BnaA03g40290D*	AT5G61520	Sugar transporter
PC3		45.08	*BnaA03g40380D*	AT5G61600	Ethylene-responsive transcription factor ERF104
PC3		136.63	*BnaA03g40560D*	AT3G49700	1-aminocyclopropane-1-carboxylate synthase
PC3	BnvaA0903085508	−4.61	*BnaA09g06240D*	AT5G62810	Peroxisomal membrane protein PEX14
PC3		0	*BnaA09g06250D*	AT5G62850	Bidirectional sugar transporter SWEET5
PC3	BnvaA0928011138	−119.96	*BnaA09g39340D*	AT3G61620	3′-5′-exoribonuclease family protein
PC3		−111.25	*BnaA09g39350D*	AT3G61630	Ethylene-responsive transcription factor CRF6
PC3		−78.77	*BnaA09g39420D*	AT3G61690	Nucleotidyltransferases
PC3		−70.32	*BnaA09g39430D*	AT3G61720	Ca^2+^ dependent phosphoribosyltransferase family protein
PC3		13.06	*BnaA09g39530D*	AT3G61960	Protein kinase superfamily protein
PC3		102.48	*BnaA09g39740D*	AT3G62190	Chaperone DnaJ-domain superfamily protein
PC3		104.85	*BnaA09g39750D*	AT3G62200	Putative endonuclease or glycosyl hydrolase
PC3		117.73	*BnaA09g39790D*	AT3G62310	RNA helicase family protein
PC3		148.6	*BnaA09g39910D*	AT3G62470	Pentatricopeptide repeat-containing protein
PC3	BnvaC0132342151	−5.7	*BnaC01g33060D*	AT3G19830	Calcium-dependent lipid-binding family protein
PC3	BnvaC0320454520	0	*BnaC03g33580D*	AT3G03960	T-complex protein
PC3		2.46	*BnaC03g33590D*	AT3G04050	Pyruvate kinase family protein
PC4	BnvaA0121360636	−81.3	*BnaA01g31290D*	AT3G11020	Dehydration-responsive element-binding protein 2B
PC4		0	*BnaA01g31380D*	AT3G10890	Mannan endo-1,4-beta-mannosidase
PC4		56.35	*BnaA01g31510D*	AT3G10680	HSP20-like chaperones superfamily protein
PC5	BnvaA0902144599	−107.17	*BnaA09g04070D*	AT5G26830	Threonine--tRNA ligase
PC5		−61.54	*BnaA09g04190D*	AT5G26742	DEAD-box ATP-dependent RNA helicase
PC5		−7.47	*BnaA09g04350D*	AT5G26200	Mitochondrial substrate carrier family protein
PC5		73.64	*BnaA09g04510D*	AT5G25620	Indole-3-pyruvate monooxygenase
PC5		84.62	*BnaA09g04520D*	AT5G25610	Dehydration-responsive protein RD22
PC5	BnvaA0500524170	−16.56	*BnaA05g00880D*	AT3G60980	Pentatricopeptide repeat-containing protein
PC5		−13.64	*BnaA05g00890D*	AT3G60960	Pentatricopeptide repeat-containing protein
PC5	BnvaC0634748907	−21.86	*BnaC06g36100D*	AT1G75310	auxin-like 1 protein
PC5		142.14	*BnaC06g36290D*	AT1G75580	SAUR-like auxin-responsive protein family

## Supplementary Materials

The following are available online at https://www.mdpi.com/2223-7747/10/3/426/s1, Figure S1: Frequency distribution of the stress tolerance indices for principal components, Figure S2: Prioritization of candidate genes around SNP BnvaA0604099845 based on comprehensive score of GWAS p-value, large effect variation and haplotype variation. Figure S3: Prioritization of candidate genes around SNP BnvaA0903085508 based on comprehensive score of GWAS p-value, large effect variation and haplotype variation. Figure S4: Prioritization of the candidate genes around SNP BnvaA0903085508 based on comprehensive score of GWAS p-value, large effect variation and haplotype variation. Figure S5: Prioritization of candidate genes around SNP BnvaC0206243256 based on comprehensive score of GWAS p-value, large effect variation and haplotype variation. Figure S6: The Genome-wide linkage disequilibrium (LD) decay in the whole genomes for the 442 accessions. Table S1: List of the 442 accessions used for genome wide association study.
