# Supplementary material for: Genome-Wide Association Mapping Unravels the Genetic Control of Seed Vigor under Low-Temperature Conditions in Rapeseed (Brassica napus L.)
Source: Plants (Basel). 2021 Feb 24;10(3):426. doi: 10.3390/plants10030426 (PMC7996214; doi:10.3390/plants10030426)
Supplement: Supplementary file 1 [file plants-10-00426-s001.zip › plants-1100247-supplementary.pdf]

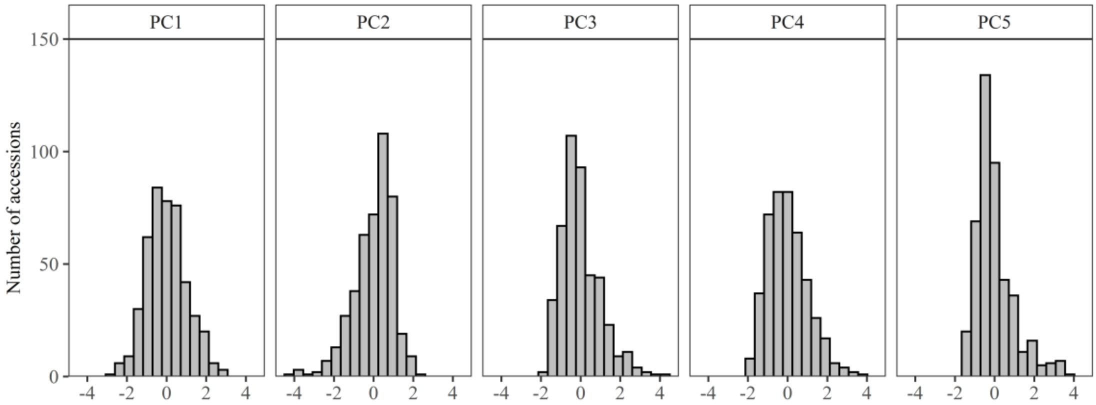

Supplementary Figure S1. Frequency distribution of the stress tolerance indices for principal components. PC1 is the comprehensive STI index of RL, TL, SVI and RGR. PC2 is the comprehensive STI index of GI, PG and MGT. PC3 is the comprehensive STI index of DWS, DWR and TDW. PC4 is the comprehensive STI index of GI, PG and MGT. PC5 is the STI index of SL. MGT, mean germination time; GI, germination index; PG, percentage of germination; MET, mean emergence time; PE, percentage of emergence; DWS, dry weight of shoot; DWR, dry weight of root; TDW, total dry weight; RL, root length; SL, shoot length; TL, total length; SVI, seedling vigor index; RGR, root growth rate.

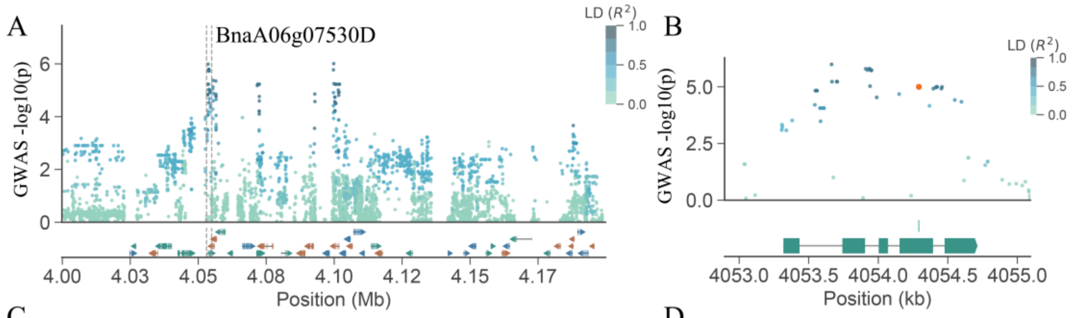

C

Gene	EV	EH	GF	SS	Description
BnaA06g07530D	0.93	1.00	0.41	1.19	RING/U-box superfamily protein
BnaA06g07550D	0.56	0.65	0.42	0.78	RNA-binding (RRM/RBD/RNP motifs) family protein
BnaA06g07860D	0.36	0.56	0.51	0.76	Transcription factor SCREAM2
BnaA06g07850D	0.32	0.57	0.52	0.75	Transcription factor SCREAM2
BnaA06g07650D	0.91	0.30	0.46	0.69	Proteasome subunit
BnaA06g07540D	0.26	0.65	0.39	0.61	RNA-binding (RRM/RBD/RNP motifs) family protein
BnaA06g07560D	0.00	0.65	0.42	0.55	Actin-related protein
BnaA06g07870D	0.26	0.52	0.35	0.46	
BnaA06g07790D	0.00	0.44	0.51	0.45	Glyceraldehyde-3-phosphate dehydrogenase
BnaA06g07600D	0.07	0.51	0.37	0.41	Cellulase (glycosyl hydrolase family) protein

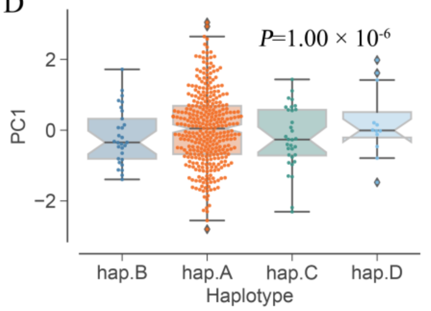

Supplementary Figure S2. Prioritization of candidate genes around SNP BnvaA0604099845 based on comprehensive score of GWAS p-value, large effect variation and haplotype variation. (A) Manhattan plot of the SNPs in the 150kbp regions flanking the candidate gene *BnaA06g07530D*, and the window of the dash lines represent the SNPs located in region of the specific gene. (B) GWAS results at the *BnaA06g07530D* gene itself with the high effect SNPs are indicated by gold circles. (C) Ranking the top ten genes according to the summary score and the top-ranked gene is the most likely casual gene. EV, the effect of variations; EH, effect of haplotypes; GF, the score of probability for gene function; SS, summary score of GF, EV, and EH. (D) Box plots displaying the impacts of haplotypes variations derived from the specific gene on the phenotype.

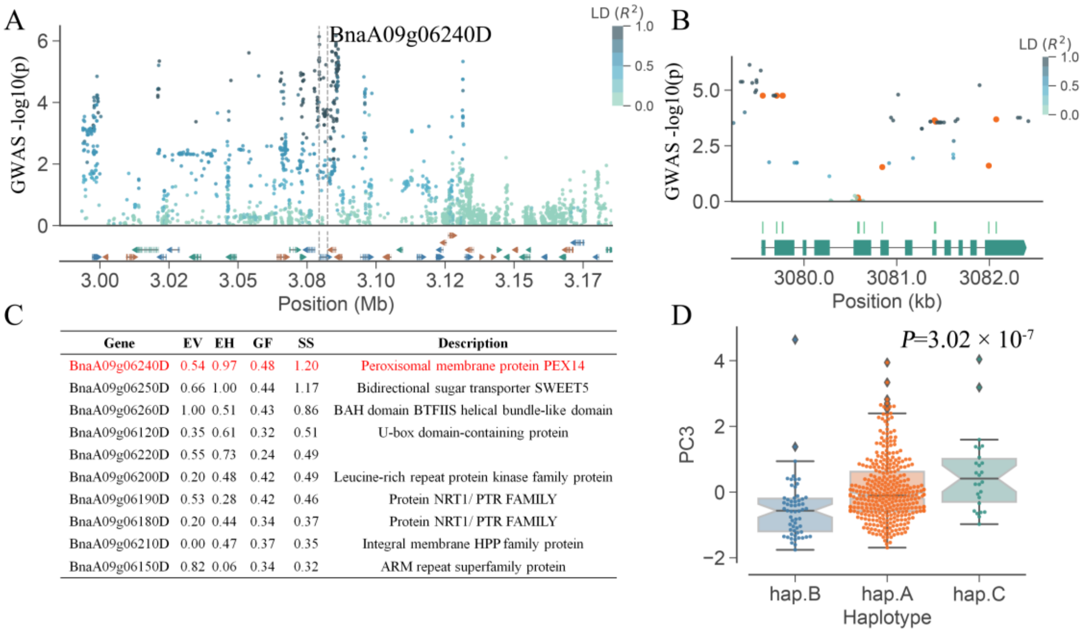

Gene	EV	EH	GF	SS	Description
BnaA09g06240D	0.54	0.97	0.48	1.20	Peroxisomal membrane protein PEX14
BnaA09g06250D	0.66	1.00	0.44	1.17	Bidirectional sugar transporter SWEET5
BnaA09g06260D	1.00	0.51	0.43	0.86	BAH domain BTFIIS helical bundle-like domain
BnaA09g06120D	0.35	0.61	0.32	0.51	U-box domain-containing protein
BnaA09g06220D	0.55	0.73	0.24	0.49	
BnaA09g06200D	0.20	0.48	0.42	0.49	Leucine-rich repeat protein kinase family protein
BnaA09g06190D	0.53	0.28	0.42	0.46	Protein NRT1/ PTR FAMILY
BnaA09g06180D	0.20	0.44	0.34	0.37	Protein NRT1/ PTR FAMILY
BnaA09g06210D	0.00	0.47	0.37	0.35	Integral membrane HPP family protein
BnaA09g06150D	0.82	0.06	0.34	0.32	ARM repeat superfamily protein

Supplementary Figure S3. Prioritization of candidate genes around SNP BnvaA0903085508 based on comprehensive score of GWAS p-value, large effect variation and haplotype variation. (A) Manhattan plot of the SNPs in the 150kbp regions flanking the candidate gene *BnaA06g07530D*, and the window of the dash lines represent the SNPs located in region of the specific gene. (B) GWAS results at the *BnaA06g07530D* gene itself with the high effect SNPs are indicated by gold circles. (C) Ranking the top ten genes according to the summary score and the top-ranked gene is the most likely casual gene. EV, the effect of variations; EH, effect of haplotypes; GF, the score of probability for gene function; SS, summary score of GF, EV, and EH. (D) Box plots displaying the impacts of haplotypes variations derived from the specific gene on the phenotype.

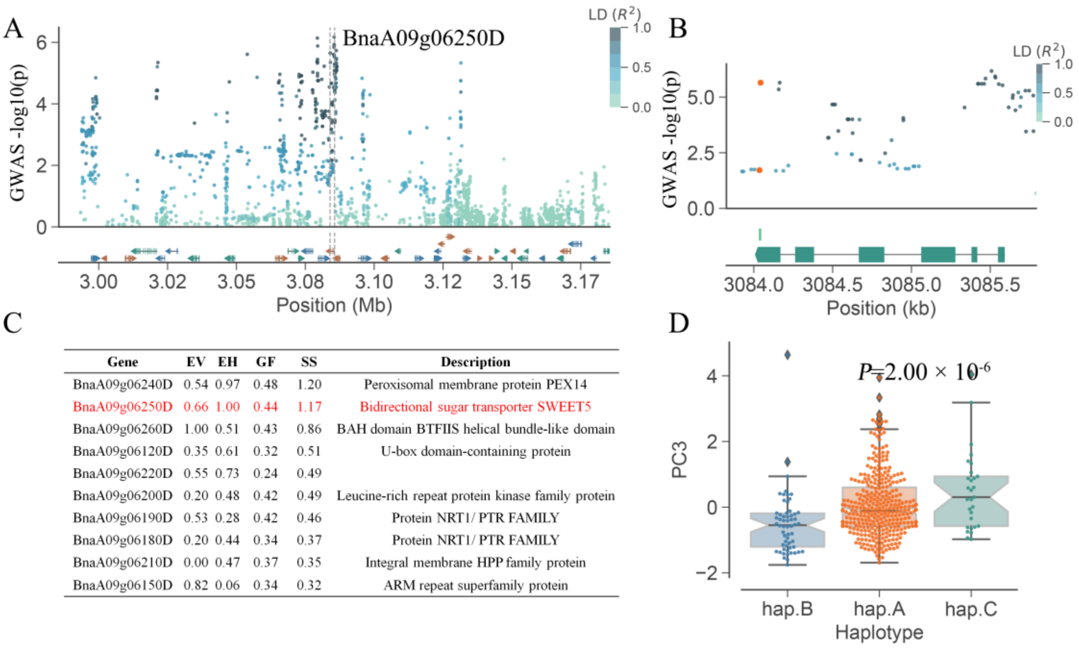

Gene	EV	EH	GF	SS	Description
BnaA09g06240D	0.54	0.97	0.48	1.20	Peroxisomal membrane protein PEX14
BnaA09g06250D	0.66	1.00	0.44	1.17	Bidirectional sugar transporter SWEET5
BnaA09g06260D	1.00	0.51	0.43	0.86	BAH domain BTFIIS helical bundle-like domain
BnaA09g06120D	0.35	0.61	0.32	0.51	U-box domain-containing protein
BnaA09g06220D	0.55	0.73	0.24	0.49	
BnaA09g06200D	0.20	0.48	0.42	0.49	Leucine-rich repeat protein kinase family protein
BnaA09g06190D	0.53	0.28	0.42	0.46	Protein NRT1/ PTR FAMILY
BnaA09g06180D	0.20	0.44	0.34	0.37	Protein NRT1/ PTR FAMILY
BnaA09g06210D	0.00	0.47	0.37	0.35	Integral membrane HPP family protein
BnaA09g06150D	0.82	0.06	0.34	0.32	ARM repeat superfamily protein

Supplementary Figure S4. Prioritization of the candidate genes around SNP BnvaA0903085508 based on comprehensive score of GWAS p-value, large effect variation and haplotype variation. (A) Manhattan plot of the SNPs in the 150kbp regions flanking the candidate gene *BnaA09g06250D*, and the window of the dash lines represent the SNPs located in region of the specific gene. (B) GWAS results at the *BnaA09g06250D* gene itself with high effect SNPs are indicated by gold circles. (C) Ranking the top ten genes according to the summary score and the top-ranked gene is the most likely casual gene. EV, the effect of variations; EH, effect of haplotypes; GF, the score of probability for gene function; SS, summary score of GF, EV, and EH. (D) Box plots displaying the impacts of haplotypes variations derived from the specific gene on the phenotype.

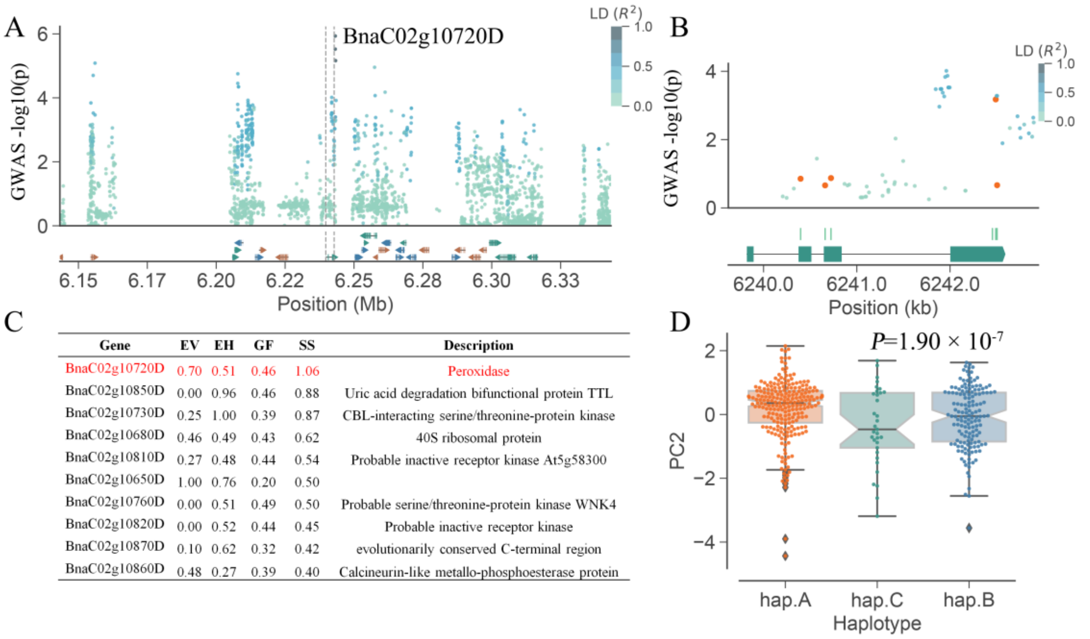

Gene	EV	EH	GF	SS	Description
BnaC02g10720D	0.70	0.51	0.46	1.06	Peroxidase
BnaC02g10850D	0.00	0.96	0.46	0.88	Uric acid degradation bifunctional protein TTL
BnaC02g10730D	0.25	1.00	0.39	0.87	CBL-interacting serine/threonine-protein kinase
BnaC02g10680D	0.46	0.49	0.43	0.62	40S ribosomal protein
BnaC02g10810D	0.27	0.48	0.44	0.54	Probable inactive receptor kinase At5g58300
BnaC02g10650D	1.00	0.76	0.20	0.50	
BnaC02g10760D	0.00	0.51	0.49	0.50	Probable serine/threonine-protein kinase WNK4
BnaC02g10820D	0.00	0.52	0.44	0.45	Probable inactive receptor kinase
BnaC02g10870D	0.10	0.62	0.32	0.42	evolutionarily conserved C-terminal region
BnaC02g10860D	0.48	0.27	0.39	0.40	Calcineurin-like metallo-phosphoesterase protein

Supplementary Figure S5. Prioritization of candidate genes around SNP BnvaC0206243256 based on comprehensive score of GWAS p-value, large effect variation and haplotype variation. (A) Manhattan plot of the SNPs in the 150kbp regions flanking the candidate gene *BnaC02g10720D*, and the window of the dash lines represent the SNPs located in region of the specific gene. (B) GWAS results at the *BnaC02g10720D* gene itself with high effect SNPs are indicated by gold circles. (C) Ranking the top ten genes according to the comprehensive scores and the top-ranked gene is the most likely casual gene. EV, the effect of variations; EH, effect of haplotypes; GF, the score of probability for gene function; SS, summary score of GF, EV, and EH. (D) Box plots displaying the impacts of haplotypes variations derived from the specific gene on the phenotype.

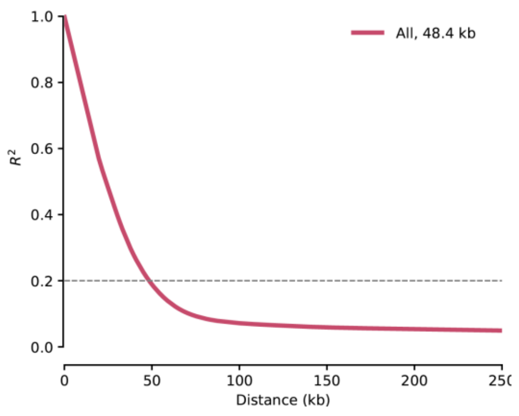

Supplementary Figure S6. The Genome-wide linkage disequilibrium (LD) decay in the whole genomes for the 442 accessions. The squared allele frequency (r^2) = 0.2 was used as the cut-off.

Supplementary Table S1. List of the 442 accessions used for genome wide association study.

Accessions	Origin	Type	Accessions	Origin	Type
A109	Shanxi (China)	Semi-winter	Swu90	Chongqing (China)	Semi-winter
A117	Shanxi (China)	Semi-winter	Swu92	Chongqing (China)	Semi-winter
A148	Sweden	Spring	Swu93	Chongqing (China)	Semi-winter
A172	Shanxi (China)	Semi-winter	Swu94	Chongqing (China)	Semi-winter
A82	Jiangxi (China)	Semi-winter	Swu95	Chongqing (China)	Semi-winter
A97	Sichuan (China)	Semi-winter	Swu96	Chongqing (China)	Semi-winter
Agrev019	German	Spring	Swu97	Chongqing (China)	Semi-winter
Agrev021	German	Spring	Swu99	Chongqing (China)	Semi-winter
Aurora	German	Spring	Taisetsu	Japan	Vegetable
B265	Shanxi (China)	Semi-winter	Tianyou4	Gansu (China)	Winter
B285	Shanxi (China)	Semi-winter	Wanyou12	Anhui (China)	Semi-winter
B414	Xinjiang (China)	Semi-winter	Wanyou15	Anhui (China)	Semi-winter
Bienvenu	France	Winter	Wanyou16	Anhui (China)	Semi-winter
Ca-318	German	Spring	Wanyou20	Anhui (China)	Semi-winter
Canadian2	Canada	Spring	Wanyou29	Anhui (China)	Semi-winter
Caoyou2	Shanghai (China)	Semi-winter	Wase Chousen	Japan	Spring
Ceres	German	Winter	We-241	Canada	Spring
Chu107	Anhui (China)	Semi-winter	Weilong88	Hubei (China)	Semi-winter
Chu610	Anhui (China)	Semi-winter	Wesreo	Australia	Spring
Chuanyou18	Sichuan (China)	Semi-winter	Wh-127	Hubei (China)	Semi-winter
Chuanyou20	Sichuan (China)	Semi-winter	Wh-15	Hubei (China)	Semi-winter
Chuyou1	Anhui (China)	Semi-winter	Wh-17	Hubei (China)	Semi-winter
Cobra	German	Winter	Wh-19	Hubei (China)	Semi-winter

Comet	Sweden	Spring	Wh-20	Hubei (China)	Semi-winter
Conny	Sweden	Spring	Wh-23	Hubei (China)	Semi-winter
Cresor	France	Spring	Wh-24	Hubei (China)	Semi-winter
Cubs Root	Korea	Spring	Wh-26	Hubei (China)	Semi-winter
Cy12Gj-1	Sichuan (China)	Semi-winter	Wh-29	Hubei (China)	Semi-winter
Cy12Ny-7	Sichuan (China)	Semi-winter	Wh-30	Hubei (China)	Semi-winter
Cy12Pxw-4	Sichuan (China)	Semi-winter	Wh-33	Hubei (China)	Semi-winter
Cy12Pxw-6	Sichuan (China)	Semi-winter	Wh-38	Hubei (China)	Semi-winter
Cy12Pxw-9	Sichuan (China)	Semi-winter	Wh-42	Hubei (China)	Semi-winter
Cy12Q21535-N3	Sichuan (China)	Semi-winter	Wh-43	Hubei (China)	Semi-winter
Cy12Q8-7	Sichuan (China)	Semi-winter	Wh-45	Hubei (China)	Semi-winter
Cy12Q95108	Sichuan (China)	Semi-winter	Wh-49	Hubei (China)	Semi-winter
Cy12Q95406	Sichuan (China)	Semi-winter	Wh-50	Hubei (China)	Semi-winter
Cy12Qcwh-1	Sichuan (China)	Semi-winter	Wh-57	Hubei (China)	Semi-winter
Cy12Qsz06	Sichuan (China)	Semi-winter	Wh-58	Hubei (China)	Semi-winter
Cy13Pxw-17	Sichuan (China)	Semi-winter	Wh-59	Hubei (China)	Semi-winter
Cy14Pxw-18	Sichuan (China)	Semi-winter	Wh-60	Hubei (China)	Semi-winter
Cy15Pxw-31	Sichuan (China)	Semi-winter	Wh-61	Hubei (China)	Semi-winter
Cy16Pxw-35	Sichuan (China)	Semi-winter	Wh-62	Hubei (China)	Semi-winter
Cy17Pxw-58	Sichuan (China)	Semi-winter	Wh-63	Hubei (China)	Semi-winter
Cy18Pxw-62	Sichuan (China)	Semi-winter	Wh-83	Hubei (China)	Semi-winter
Cy19Pxw-65	Sichuan (China)	Semi-winter	Wh-85	Hubei (China)	Semi-winter
Cy20Pxw-66	Sichuan (China)	Semi-winter	Wh-88	Hubei (China)	Semi-winter
Cy21Pxw-84	Sichuan (China)	Semi-winter	Wh-95	Hubei (China)	Semi-winter

D2	Danmark	Spring	Wu164	Gansu (China)	Spring
Daichousen	Japan	Semi-winter	Wx1025	Hunan (China)	Semi-winter
De68-12	Anhui (China)	Semi-winter	Wx10296	Hunan (China)	Semi-winter
Enyou73-1-2	Chongqing (China)	Semi-winter	Wx10315	Hunan (China)	Semi-winter
Erake	Poland	Spring	Wx10329	Hunan (China)	Semi-winter
Fengyou9	Henan (China)	Semi-winter	Xiangyou11	Hunan (China)	Semi-winter
FTD1	Hubei (China)	Semi-winter	Xiangyou13	Hunan (China)	Semi-winter
FTD2	Hubei (China)	Semi-winter	Xiangyou15	Hunan (China)	Semi-winter
FTD3	Hubei (China)	Semi-winter	Xiwang106	Hubei (China)	Semi-winter
FTD4	Hubei (China)	Semi-winter	Y1	Shanghai (China)	Semi-winter
FTD5	Hubei (China)	Semi-winter	Y14	Jiangsu (China)	Semi-winter
FTD6	Hubei (China)	Semi-winter	Y16	Jiangsu (China)	Semi-winter
FTD7	Hubei (China)	Semi-winter	Y2	Shanghai (China)	Semi-winter
FTD8	Hubei (China)	Semi-winter	Y3	Shanghai (China)	Semi-winter
Fuyou4	Heilongjiang(China)	Spring	Y32	Jiangsu (China)	Semi-winter
Ganyou5	Hubei (China)	Semi-winter	Y33	Japan	Semi-winter
Gl302-1	Gansu (China)	Spring	Y38	Anhui (China)	Semi-winter
Guangde138	Anhui (China)	Semi-winter	Y4	Shanghai (China)	Semi-winter
Guangde761	Anhui (China)	Semi-winter	Y40	Anhui (China)	Semi-winter
Guangde8104	Anhui (China)	Semi-winter	Y44	Anhui (China)	Semi-winter
Gy270	Shanxi (China)	Semi-winter	Y46	Anhui (China)	Semi-winter
Gy282	Shanxi (China)	Semi-winter	Y6	Shanghai (China)	Semi-winter
H-6	Japan	Semi-winter	Y8	Shanghai (China)	Semi-winter
Hai-244	Canada	Spring	Yan81-2	Chongqing (China)	Semi-winter

He-426	Hubei (China)	Semi-winter	Yangguang198	Hubei (China)	Semi-winter
Hongyou3	Jiangsu (China)	Semi-winter	Yangguang2009	Hubei (China)	Semi-winter
Huahang901	Hubei (China)	Semi-winter	Yangguang209	Hubei (China)	Semi-winter
Huashuang128	Hubei (China)	Semi-winter	Yangj6711	Jiangsu (China)	Semi-winter
Huashuang2	Hubei (China)	Semi-winter	Yangyou4	Jiangsu (China)	Semi-winter
Huashuang4	Hubei (China)	Semi-winter	Yangyou5	Jiangsu (China)	Semi-winter
Huashuang5	Hubei (China)	Semi-winter	Yanyou2	Jiangsu (China)	Semi-winter
Huayou12	Hubei (China)	Semi-winter	Yb3	Hunan (China)	Semi-winter
Huayou13	Hubei (China)	Semi-winter	Youyan2	Guizhou (China)	Semi-winter
Huayou14	Hubei (China)	Semi-winter	Yuyou1	Henan (China)	Semi-winter
Huayou2	Hubei (China)	Semi-winter	Zaofeng1	Zhejiang (China)	Semi-winter
Huayou3	Hubei (China)	Semi-winter	Zh-243	Canada	Spring
Huayou4	Hubei (China)	Semi-winter	Zhen3736	Jiangsu (China)	Semi-winter
Huayou6	Hubei (China)	Semi-winter	Zhengyou3	Jiangsu (China)	Semi-winter
Huyou12	Shanghai (China)	Semi-winter	Zhenyou5	Jiangsu (China)	Semi-winter
Huyou14	Shanghai (China)	Semi-winter	Zhenyou6	Jiangsu (China)	Semi-winter
Huyou15	Shanghai (China)	Semi-winter	Zheshuang3	Zhejiang (China)	Semi-winter
Huyou17	Shanghai (China)	Semi-winter	Zheshuang6	Zhejiang (China)	Semi-winter
Huyou18	Shanghai (China)	Semi-winter	Zheshuang72	Zhejiang (China)	Semi-winter
Huyou19	Shanghai (China)	Semi-winter	Zheshuang8	Zhejiang (China)	Semi-winter
Huyou21	Shanghai (China)	Semi-winter	Zheyou17	Zhejiang (China)	Semi-winter
Huyou3	Shanghai (China)	Semi-winter	Zheyou18	Zhejiang (China)	Semi-winter

Imc103	Chongqing (China)	Semi-winter	Zheyou19	Zhejiang (China)	Semi-winter
Jia904	Hubei (China)	Semi-winter	Zheyou21	Zhejiang (China)	Semi-winter
Jia908	Hubei (China)	Semi-winter	Zheyou50	Zhejiang (China)	Semi-winter
Jia915	Hubei (China)	Semi-winter	Zheyou601	Zhejiang (China)	Semi-winter
Jia917	Hubei (China)	Semi-winter	Zheyou758	Zhejiang (China)	Semi-winter
Jia922	Hubei (China)	Semi-winter	Zhongshuang10	Hubei (China)	Semi-winter
Jia923	Hubei (China)	Semi-winter	Zhongshuang11	Hubei (China)	Semi-winter
Jia931	Hubei (China)	Semi-winter	Zhongshuang12	Hubei (China)	Semi-winter
Jia951	Hubei (China)	Semi-winter	Zhongshuang2	Hubei (China)	Semi-winter
Jia963	Hubei (China)	Semi-winter	Zhongshuang3	Hubei (China)	Semi-winter
Jiapf190	Hubei (China)	Semi-winter	Zhongshuang4	Hubei (China)	Semi-winter
Jiayu05	Hubei (China)	Semi-winter	Zhongshuang6	Hubei (China)	Semi-winter
Jiayu16	Hubei (China)	Semi-winter	Zhongshuang7	Hubei (China)	Semi-winter
Jiayu17	Hubei (China)	Semi-winter	Zhongshuang9	Hubei (China)	Semi-winter
Jiayu25	Hubei (China)	Semi-winter	Zhongyou589	Hubei (China)	Semi-winter
Jiayu31	Hubei (China)	Semi-winter	Zhongyou821	Hubei (China)	Semi-winter
Jie65-1	Zhejiang (China)	Semi-winter	509	Hunan (China)	Semi-winter
Kelina	Chongqing (China)	Semi-winter	563	Hunan (China)	Semi-winter
Kenc1	Shanxi (China)	Semi-winter	631	Hunan (China)	Semi-winter
L134	Hubei (China)	Semi-winter	699	Hubei (China)	Semi-winter
L135	Hubei (China)	Semi-winter	782	Hunan (China)	Semi-winter
L136	Hubei (China)	Semi-winter	783	Hunan (China)	Semi-winter

L137	Hubei (China)	Semi-winter	876	Gansu (China)	Winter
L138	Hubei (China)	Semi-winter	986	Gansu (China)	Winter
L139	Hubei (China)	Semi-winter	1111	Hubei (China)	Semi-winter
L140	Hubei (China)	Semi-winter	1188	Hubei (China)	Semi-winter
L385	Zhejiang (China)	Semi-winter	1252	Hunan (China)	Semi-winter
L389	Jiangsu (China)	Semi-winter	1281	Hunan (China)	Semi-winter
L392	Shanghai (China)	Semi-winter	1321	Hunan (China)	Semi-winter
L394	Shanghai (China)	Semi-winter	1322	Hunan (China)	Semi-winter
L395	Shanghai (China)	Semi-winter	1360	Hunan (China)	Semi-winter
L396	Zhejiang (China)	Semi-winter	1368	Hunan (China)	Semi-winter
L397	Zhejiang (China)	Semi-winter	1472	Hunan (China)	Semi-winter
L400	Hubei (China)	Semi-winter	1570	Hubei (China)	Semi-winter
L401	Hubei (China)	Semi-winter	2354	Hubei (China)	Semi-winter
L402	Hubei (China)	Semi-winter	2359	Hubei (China)	Semi-winter
L407	Hubei (China)	Semi-winter	2365	Hubei (China)	Semi-winter
L503	Shanghai (China)	Semi-winter	7016	Hubei (China)	Semi-winter
L508	Jiangsu (China)	Semi-winter	7022	Hubei (China)	Semi-winter
Longyou2	Gansu (China)	Spring	7037	Hubei (China)	Semi-winter
Longyou4	Gansu (China)	Spring	7094	Hubei (China)	Semi-winter
Major	France	Winter	7189	Hubei (China)	Semi-winter
Monty	Australia	Spring_	7191	Hubei (China)	Semi-winter
Nakaee Chousen	Japan	Spring	9852	Gansu (China)	Winter
Nca	Hubei (China)	Semi-winter	23651	Hubei (China)	Semi-winter
Niklas	Denmark	Spring	28887	Chongqing (China)	Semi-winter

Ningyou 7	Jiangsu (China)	Semi-winter	42645	Hubei (China)	Semi-winter
Ningyou1	Jiangsu(China)	Semi-winter	42646	Hubei (China)	Semi-winter
Ningyou12	Jiangsu (China)	Semi-winter	42666	Hubei (China)	Semi-winter
Ningyou18	Jiangsu (China)	Semi-winter	42667	Hubei (China)	Semi-winter
Ningyou6	Jiangsu (China)	Semi-winter	42668	Hubei (China)	Semi-winter
Ningyou8	Jiangsu (China)	Semi-winter	42672	Hubei (China)	Semi-winter
Niuerduo	Chongqing (China)	Semi-winter	90750	Chongqing (China)	Semi-winter
Norin42	Japan	Semi-winter	93210	Hubei (China)	Semi-winter
Norin43	Japan	Semi-winter	96021	Hubei (China)	Semi-winter
O-136	Chongqing (China)	Semi-winter	96063	Hubei (China)	Semi-winter
P113	Shanxi (China)	Semi-winter	97096	Hubei (China)	Semi-winter
P18	Hubei (China)	Semi-winter	97097	Hubei (China)	Semi-winter
P310	Shanxi (China)	Semi-winter	97177	Hubei (China)	Semi-winter
P668	Shanxi (China)	Semi-winter	880101	Chongqing (China)	Semi-winter
P685	Shanxi (China)	Semi-winter	03I32B	Gansu (China)	Spring
Peixuan170	Jiangsu (China)	Semi-winter	03II3B	Gansu (China)	Spring
Q-247	the USA	Spring	03II4B	Gansu (China)	Spring
Qianyou331	Guizhou (China)	Semi-winter	03Lf1	Gansu (China)	Spring
Qianyou4	Guizhou (China)	Semi-winter	06H7	Gansu (China)	Spring
Qing662	Hubei (China)	Semi-winter	06T9F	Gansu (China)	Spring
Qinyou1	Shanxi (China)	Semi-winter	08-P35	Hubei (China)	Semi-winter
Qinyou5	Shanxi (China)	Semi-winter	08-P36	Hubei (China)	Semi-winter

R2	Hubei (China)	Semi-winter	09-P36	Hubei (China)	Semi-winter
Rq011	Australia	Spring	09-P37	Hubei (China)	Semi-winter
Rr002	Australia	Spring	10-1043	Hunan (China)	Semi-winter
Rr009	Australia	Spring	10-1047	Hunan (China)	Semi-winter
Rucabo	German	Spring	10-1061	Hunan (China)	Semi-winter
Santana	German	Winter	10-1070	Hunan (China)	Semi-winter
Shan2B	Shanxi (China)	Semi-winter	10-1230	Qinghai (China)	Spring
Shengguang77	Hubei (China)	Semi-winter	10-1358	Hunan (China)	Semi-winter
Shengliyoucai	Japan	Semi-winter	10-32	Hubei (China)	Semi-winter
Shenhuang1	Shanghai (China)	Semi-winter	10-33	Hubei (China)	Semi-winter
Shilifeng	Jiangsu (China)	Semi-winter	10-34	Hubei (China)	Semi-winter
Shilijia	Jiangsu (China)	Semi-winter	10-758	Qinghai (China)	Spring
Si-242	Canada	Spring	10-804	Hunan (China)	Semi-winter
Sophia	unknown	Spring	10-P10	Hubei (China)	Semi-winter
Suyou1	Jiangsu (China)	Semi-winter	10-P29	Hubei (China)	Semi-winter
Suyou4	Jiangsu (China)	Semi-winter	11-1184	Qinghai (China)	Spring
Swu01	Hubei (China)	Semi-winter	11-504	Qinghai (China)	Spring
Swu100	Chongqing (China)	Semi-winter	11-540	Qinghai (China)	Spring
Swu102	Chongqing (China)	Semi-winter	11-7-103	Hubei (China)	Semi-winter
Swu103	Chongqing (China)	Semi-winter	11-7-117	Hubei (China)	Semi-winter
Swu104	Chongqing (China)	Semi-winter	11-7-125	Hubei (China)	Semi-winter
Swu106	Chongqing (China)	Semi-winter	11-9-700	Hubei (China)	Semi-winter
Swu110	Chongqing (China)	Semi-winter	11-9-701	Hubei (China)	Semi-winter
Swu111	Chongqing (China)	Semi-winter	11-9-703	Hubei (China)	Semi-winter
Swu113	Chongqing (China)	Semi-winter	11-9-704	Hubei (China)	Semi-winter

Swu114	Chongqing (China)	Semi-winter	11-9-705	Hubei (China)	Semi-winter
Swu39	Sichuan (China)	Semi-winter	11-9-706	Hubei (China)	Semi-winter
Swu40	Chongqing (China)	Semi-winter	11-9-707	Hubei (China)	Semi-winter
Swu41	Chongqing (China)	Semi-winter	11-997	Qinghai (China)	Spring
Swu42	Chongqing (China)	Semi-winter	11-P30	Hubei (China)	Semi-winter
Swu43	Chongqing (China)	Semi-winter	11-P63-5	Hubei (China)	Semi-winter
Swu44	Chongqing (China)	Semi-winter	11-P63-8	Hubei (China)	Semi-winter
Swu45	Chongqing (China)	Semi-winter	11-P67	Hubei (China)	Semi-winter
Swu46	Chongqing (China)	Semi-winter	11-P74-13	Hubei (China)	Semi-winter
Swu47	Chongqing (China)	Semi-winter	11-P74-8	Hubei (China)	Semi-winter
Swu48	Chongqing (China)	Semi-winter	12-P24	Hubei (China)	Semi-winter
Swu49	Chongqing (China)	Semi-winter	12-P25	Hubei (China)	Semi-winter
Swu52	Chongqing (China)	Semi-winter	2011-6200	Hubei (China)	Semi-winter
Swu53	Chongqing (China)	Semi-winter	2011-6308	Hubei (China)	Semi-winter
Swu54	Chongqing (China)	Semi-winter	2011-7103	Hubei (China)	Semi-winter
Swu56	Chongqing (China)	Semi-winter	2012-11526	Hubei (China)	Semi-winter
Swu57	Chongqing (China)	Semi-winter	2012-3448	Hubei (China)	Semi-winter
Swu59	Chongqing (China)	Semi-winter	2012-3546	Hubei (China)	Semi-winter
Swu60	Chongqing (China)	Semi-winter	2012-4531	Hubei (China)	Semi-winter
Swu61	Chongqing (China)	Semi-winter	2012-5086	Hubei (China)	Semi-winter
Swu63	Chongqing (China)	Semi-winter	2012-5113	Hubei (China)	Semi-winter
Swu65	Chongqing (China)	Semi-winter	2012-8327	Hubei (China)	Semi-winter

Swu66	Chongqing (China)	Semi-winter	2012-8355	Hubei (China)	Semi-winter
Swu68	Chongqing (China)	Semi-winter	2012-8380	Hubei (China)	Semi-winter
Swu69	Chongqing (China)	Semi-winter	2012-8998	Hubei (China)	Semi-winter
Swu70	Chongqing (China)	Semi-winter	2012-9323	Hubei (China)	Semi-winter
Swu74	Chongqing (China)	Semi-winter	2012-9354	Hubei (China)	Semi-winter
Swu75	Chongqing (China)	Semi-winter	2012-9380	Hubei (China)	Semi-winter
Swu76	Chongqing (China)	Semi-winter	2012-9478	Hubei (China)	Semi-winter
Swu77	Chongqing (China)	Semi-winter	2012-9542	Hubei (China)	Semi-winter
Swu80	Chongqing (China)	Semi-winter	2012-K8053	Hubei (China)	Semi-winter
Swu81	Chongqing (China)	Semi-winter	7-7766-74	Hubei (China)	Semi-winter
SWU82	Chongqing (China)	Semi-winter	96—203	Qinghai (China)	Semi-winter
Swu84	Chongqing (China)	Semi-winter	9Bao22	Hubei (China)	Semi-winter
Swu85	Chongqing (China)	Semi-winter	9F087	Hubei (China)	Semi-winter
